# Dynamic Response of Ammonia-Oxidizers to Four Fertilization Regimes across a Wheat-Rice Rotation System

**DOI:** 10.3389/fmicb.2017.00630

**Published:** 2017-04-12

**Authors:** Jichen Wang, Lei Ni, Yang Song, Geoff Rhodes, Jing Li, Qiwei Huang, Qirong Shen

**Affiliations:** ^1^Jiangsu Provincial Key Lab and Coordinated Research Center for Organic Solid Waste Utilization, Nanjing Agricultural UniversityNanjing, China; ^2^Department of Plant, Soil and Microbial Sciences, Michigan State UniversityEast Lansing, MI, USA

**Keywords:** ammonia-oxidizers community, nitrification, fertilization regime, dynamic variation, wheat-rice rotation system

## Abstract

Ammonia oxidation by microorganisms is a rate-limiting step of the nitrification process and determines the efficiency of fertilizer utilized by crops. Little is known about the dynamic response of ammonia-oxidizers to different fertilization regimes in a wheat-rice rotation system. Here, we examined ammonia-oxidizing bacteria (AOB) and archaea (AOA) communities across eight representative stages of wheat and rice growth and under four fertilization regimes: no nitrogen fertilization (NNF), chemical fertilization (CF), organic-inorganic mixed fertilizer (OIMF) and organic fertilization (OF). The abundance and composition of ammonia oxidizers were analyzed using quantitative PCR (qPCR) and terminal restriction fragment length polymorphism (T-RFLP) of their *amoA* genes. Results showed that fertilization but not plant growth stages was the best predictor of soil AOB community abundance and composition. Soils fertilized with more urea-N had higher AOB abundance, while organic-N input showed little effect on AOB abundance. 109 bp T-RF (*Nitrosospira* Cluster 3b) and 280 bp T-RF (*Nitrosospira* Cluster 3c) dominated the AOB communities with opposing responses to fertilization regimes. Although the abundance and composition of the AOA community was significantly impacted by fertilization and plant growth stage, it differed from the AOB community in that there was no particular trend. In addition, across the whole wheat-rice rotation stages, results of multiple stepwise linear regression revealed that AOB played a more important role in ammonia oxidizing process than AOA. This study provided insight into the dynamic effects of fertilization strategies on the abundance and composition of ammonia-oxidizers communities, and also offered insights into the potential of managing nitrogen for sustainable agricultural productivity with respect to soil ammonia-oxidizers.

## Introduction

Ammonia oxidation by microorganisms is the first step of nitrification. It is a rate-limiting step of the nitrification process and influences the efficiency of fertilizer utilized by crops (Kowalchuk and Stephen, 2001). Traditionally, ammonia-oxidizing bacteria (AOB) of the β- and γ- subclasses of Proteobacteria were thought to be the exclusively contributor to this key process. However, this view was challenged by the discovery of the function and transcription of the *amoA* gene in archaeal ammonia-oxidizer (AOA) (Könneke et al., 2005; Treusch et al., 2005; Prosser and Nicol, 2008). In fact, further research revealed that the abundance of AOA was higher than the abundance of AOB in most of soils (Leininger et al., 2006; He et al., 2007; Shen et al., 2008). Although AOA are more abundant in most soils, and have the capabilities for mixotrophic growth, AOB do have significantly higher specific cell activity (Prosser and Nicol, 2012). Therefore, the functional importance of AOA and AOB in different environmental conditions tends to be disparate. AOA might play a dominant role in the nitrification process in soils with low ammonia content, high acidity, or low oxygen conditions (Zhang et al., 2010, 2012), while AOB may contribute more to the nitrification process in neutral of alkaline soils, or in soils with a higher ammonia content (Shen et al., 2012; Ke et al., 2013).

Ammonia-oxidizing bacteria (AOB) are sensitive to changes in soil conditions and are considered as an indicator of soil disturbance (Kowalchuk and Stephen, 2001; Ceccherini et al., 2007; e Silva et al., 2013). In most arable soils, AOB are dominated by *Nitrosospira* Clusters 2, 3, and 4 (Prosser and Nicol, 2008), and Cluster 3 is in generally ubiquitous in soil (Fierer et al., 2009; Ai et al., 2013). Different phylotypes of AOB generally had different response to the fertilizer inputs (Bruns et al., 1999; Chu et al., 2007, 2009). In terms of AOA, although not as sensitive as AOB, there were also lots of researches reported that AOA abundance could be changed by different fertilization regimes in arable soils (Chu et al., 2008; Kelly et al., 2011; Peng et al., 2013). As reviewed by Zhalnina et al. (2012), soils with higher organic matter should be expected to have a higher AOA abundance (Stopnišek et al., 2010), and organic fertilizer treatments tended to have higher archaeal 16S rRNA abundance. Xu et al. (2012) further revealed that addition of root extract as an organic amendment to the AOA enrichment culture could increase the abundance of 1.1b clade.

Winter wheat and summer rice rotation cultivation patterns provided a critical source of food security for hundreds of millions of people (Dawe et al., 2004). In addition, due to frequent oxic/anoxic alternations caused by water management and the rice rhizosphere, this rotation system may serve as a model ecosystem for analyzing the ecology of microbial communities and biogeochemical processes (Liesack et al., 2000; Noll et al., 2005). For decades, nitrogen (N) fertilizer inputs significantly changed the soil ammonia-oxidizers of this ecosystem. As reviewed previously (Shen et al., 2012), the abundance and composition of AOB communities, but not AOA communities, were significantly changed by different fertilization treatments in most Chinese paddy soils. AOA communities were also more responsive to fertilization at low pH conditions (Chen et al., 2011; Shen et al., 2012). In addition, multiple environmental conditions varied with the wheat-rice rotation stages, which also influenced the abundance and composition of ammonia-oxidizer communities (Erguder et al., 2009). Of these varied environmental conditions, soil temperature (Tourna et al., 2008), different root exudates of wheat and rice (Chen et al., 2008) and the waterlogged management of rice crop (Fujii et al., 2010) were particularly important. Nevertheless, little is known about the dynamic changes of ammonia-oxidizers communities considering both plant growth stage and fertilization regime in the wheat-rice rotation system. Particularly, the respective contributions of AOA and AOB to nitrification during the entire wheat-rice rotation seasons remain unclear.

Therefore, we studied the abundance and composition of ammonia-oxidizer populations based on long-term fertilization of experimental fields over the course of eight seasons during a wheat-rice rotation. We also wanted to determine which played more important role of AOB and AOA in the ammonia oxidization process during a wheat-rice rotation stages. Real-time qPCR and T-RFLP fingerprinting combined with clone libraries targeting *amoA* genes were used to characterize the AOB and AOA communities.

## Materials and methods

### Sample collection and soil characterization

Soil samples were collected from a long-term experimental field as described in our previous studies (Wang et al., 2016a,b). Briefly, four fertilization regimes with three replicates including (1) no N fertilizer (NNF) with application of application of 750 kg ha^−1^ superphosphate (12% P_2_O_5_) and 183 kg ha^−1^ potassium chloride (60% K_2_O); (2) NPK chemical fertilizer (CF) with application of 391 kg ha^−1^ urea (46% N), 750 kg ha^−1^ superphosphate and 183 kg ha^−1^ potassium chloride; (3) organic-inorganic mixed fertilizer (OIMF) with application of 1500 kg ha^−1^ OIMF (11.0% organic C, 12.0% total N, 4.1% P_2_O_5_, and 4.1% K_2_O), and with an extra application of 28.5 kg ha^−1^ superphosphate and 48.5 kg ha^−1^ potassium chloride to reach total quantities of N, P_2_O_5_ and K_2_O that were equal to the CF plots; (4) organic fertilizer (OF) with 4500 kg ha^−1^ pure organic fertilizer (26.4% organic C, 2.5% total N, 1.6% P_2_O_5_, and 1.3% K_2_O, made of composted rice straw and pig manure by Tianniang Ltd. of Changshu, China), which had less N inputs than that in CF and OIMF treatments. All fertilizers were applied as basal fertilizers twice in October 25th 2012 and June 4th 2013.

Soil samples were collected to a depth of 0–20 cm in 2013 on March 1st (Mar, wheat tillering), April 1st (Apr, wheat jointing), May 1st (May, wheat heading), June 4th (Jun, wheat ripening), July 7th (Jul, rice tillering), August 16th (Aug, rice jointing), September 15th (Sep, rice heading), and October 31st (Oct, rice ripening). 4 cores (5 cm in diameter) of soil samples were randomly collected at each plot (6 × 7 m in size). The soil samples were sieved to remove the plant materials, roots and stones. Additional details about the field sites and soil collection can be found in our previous study (Wang et al., 2016a,b).

The data of following soil characteristics were collected from our previous study (Wang et al., 2016a) and used in the subsequent statistical analyses: soil organic carbon content (SOC), soil moisture, soil nitrate (${\text{NO}}_{3}^{-}$) and ammonium (NH_4_+) contents, soil available potassium content (AK), soil available phosphorus content (AP), soil total N content (TN), soil pH and electrical conductivity (EC). These data were also shown in Table S1. The details on the analysis of soil properties can be found in previous studies (Wang et al., 2016a).

Chlorate inhibition method (Kurola et al., 2005) was used to measure soil potential nitrification rate (PNR) with minor modifications: 5.0 g fresh soil was agitated in 20 ml of phosphate buffer solution (NaCl, 8.0 g L^−1^; KCl, 0.2 g L^−1^; Na_2_HPO_4_, 0.2 g L^−1^; NaH_2_PO_4_, 0.2 g L^−1^; pH 7.4) with 1 mM (NH_4_)_2_SO_4_. To inhibit nitrite oxidation, a final concentration of 10 mM potassium chlorate was added to the tube. After incubating the suspension for 24 h on a rotary shaker at 25°C and 170 rpm, ${\text{NO}}_{2}^{-}$-N was extracted by 5 ml of 2 M KCl and determined by a spectrophotometrically at 540 nm with N-(1-naphthyl) ethylenediamine dihydrochloride.

### DNA extraction and quantitative real-time PCR assay

The Methods of DNA extraction and qPCR of the AOB and AOA *amoA* genes were described previously (Wang et al., 2014b). Briefly, qPCR reactions were performed using the SYBR® Premix Ex TaqTM (Perfect Real Time) kit (TaKaRa Biotechnology Co., Dalian, China). Each reaction containing 12.5 μL of SYBR® Premix Ex TaqTM (2 ×, Takara), 0.5 μL ROX Reference dye II (50 ×, TaKaRa), 10 μL dd H_2_O, 0.5 μL (5 μM) of each primer and 1 μL (~10–30 ng) DNA template. The *amoA* gene primers of AOB were amoA-1F (5′-GGGGTTTCTACTGGTGGT-3′) and amoA-2R (5′-CCCCTCKGSAAAGCCTTCTTC-3′) and yielded a fragment of 491 bp in length (McTavish et al., 1993), the *amoA* gene primers of AOA were Arch-amoAF (5′-STAATGGTCTGGCTTAGACG-3′) and Arch-amoAR (5′-GCGGCCATCCATCTGTATGT-3′) and yield a fragment of 635 bp in length (Francis et al., 2005). The PCR protocol included an initial activation step at 95°C for 30 s, and then 40 cycles at 95°C for 5 s, and 34 s at 60°C. PCR products of AOB and AOA *amoA* gene fragments from soil samples were used to insert into PMD-19 plasmid, respectively, and the right genes inserts were chosen after sequencing and BLAST in GenBank on the NCBI's homepage (https://blast.ncbi.nlm.nih.gov/Blast.cgi) to serve as standards and positive controls. The standard plasmids were quantified using a Nanodrop ND-2000 UV–Vis Spectrophotometer (NanoDrop Technologies, Wilmington, DE, USA). Standard curves were developed by serially diluting plasmid to the final concentrations of 10^8^–10^2^ gene copies/μL. The standards and the DNA samples were performed on the same plate. A negative control was always run with water as template instead of DNA sample. The qPCR was performed in duplicates and the efficiencies of qPCR ranged from 90 to 110%, and the *R*^2^-values of the standards were higher than 0.99. CT (Cycle Threshold) values were used to calculate the numbers of AOB and AOA amoA gene abundance according to Harter et al. (2014). The possible PCR inhibitors of the DNA samples were determined by serial dilutions, and no severe inhibition was found.

### T-RFLP analysis of bacterial and archaeal *amoA* genes

The T-RFLP analysis of the *amoA* genes were used to analyze both bacterial and archaeal ammonia-oxidizers community composition. The PCR reaction was performed in a 25 μL volume containing 10.5 μL of ddH_2_O, 12.5 μL of Premix Taq™ (2×) Version 2.0 (TaKaRa), 1 μL DNA template (~10–30 ng), 0.5 μL forward primer (5 μM) labeled with 6-FAM (6-carboxyfluorescein) at the 5′ end, 0.5 μL reverse primer (5 μM). The same primers of qPCR were used in the T-RFLP analyses. The PCR protocols for both bacterial and archaeal *amoA* genes were replicated three times using the following programs: 5 min at 94°C for initial denaturing, followed by 33 cycles of 94°C for 30 s, 55°C for 45 s, and 72°C for 45 s with the final extension for 10 min at 72°C. After amplification, the triplicate PCR reactions were pooled and purified using the PCR cleanup Kit (Axygen Biosciences, Union City, CA, USA). The purification PCR products were digested with 10 units of restriction enzyme *MboI* (TaKaRa) at 37°C for 6 h and then denatured at 80°C for 10 min. Capillary electrophoresis was used to separate samples and then the precise lengths of the T-RFs were estimated, the injection time for the samples was set to 15 s at a voltage of 1.5 kV, and the separation of the fragments was done by capillary electrophoresis at 8.5 kV on a 36 cm array for 1 h by ABI 3730xl DNA Analyzer (Applied Biosystems). GeneMapper 4.0 software (Applied Biosystems) was used to generate the T-RFLP profiles.

The T-RFLP lengths ranged from 50 to 500 bp in size were considered for the further analyses. The relative abundance of the T-RFs at ± 1 bp were calculated as the percentages of total peak area in the T-RFLP profile to normalize the variation among samples caused by the DNA loading amounts to the capillary, and the T-RFs with relative abundance <1% were excluded from the further analyses.

### Clone, sequencing, and phylogenetic analysis of bacterial and archaeal *amoA* genes

In order to identify the main T-RFs of the AOB and AOA T-RFLP profiles, bacterial and archaeal *amoA* genes clone libraries were constructed using the same primers as T-RFLP but without 6-FAM labeled. Soil samples were random chosen to establish clone libraries. The purified PCR products were cloned into the PMD19-T plasmid and introduced into competent *E. coli* Top 10 cells. Totally 74 clones of AOB and 67 clones of AOA were sequenced using ABI PRISM 3730 sequencer (Applied Biosystems). The obtained sequences with more than 90% identity with each other were grouped into the same operational taxonomic unit (OTU) using Mothur (Schloss et al., 2009). Only one representative sequence of each OTU was compared using BLAST. Typical sequence in our T-RFLP experiment and their related sequences obtained by BLAST were chosen to construct the neighbor-joining tree using MEGA version 6.0 (Tamura et al., 2013). The virtual digests with *MboI* were carried out on the sequences retrieved from the clone libraries to allow the assignment of phylogenetic identity to individual T-RFs. As the sizes of T-RFs labeled with 6FAM can be underestimated (Schütte et al., 2008), we further referred to the clone library of T-RF sizes.

The sequences obtained in this study have deposited in GenBank under the Accession Numbers KX137040 to KX137113 for AOB *amoA* gene and KY501152 to KY501218 for AOA *amoA* gene.

### Statistical analyses

The effects of crops growth stages and fertilization regime and their interactions on the abundance of bacterial and archaeal *amoA* genes (log_10_-transformed to normalize the data) and soil PNR were performed with two-way analysis of variance (ANOVA). Because the interactions were significant (*P* < 0.05), the Tukey's HSD test (one-way ANOVA) was performed eight times to determine the effects of different fertilization regimes on PNR and the abundance of bacterial and archaeal *amoA* genes within each sampling time, letters not shared across columns within one sampling time indicate significant differences (*P* < 0.05). The relationships of *amoA* gene copies (log_10_-transformed) and soil properties were performed using Spearman correlation analyses. The multiple stepwise linear regression was performed to determine the variation of PNR that explained by AOB and AOA and all the measured soil parameters. ANOVAs, correlation analyses and multiple stepwise linear regression were performed using SPSS 17.0.

To determine the influence of crop's growth stages and fertilization regime on the T-RFLP profiles of both bacterial and archaeal *amoA* genes, permutational multivariate analysis was performed. Redundancy analysis (RDA) was performed to analyze the relationships between soil properties and T-RFLP profiles of bacterial and archaeal *amoA* genes, and followed by 999 permutations to test the significance. The permutational multivariate analyses and RDA were performed in R software (Team, 2014) with the package “vegan.”

## Results

### Soil PNR

**Figure 1:**
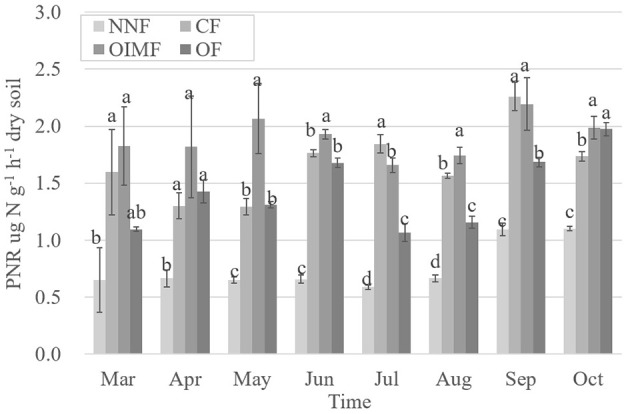
**Dynamic changes of soil potential nitrification rate (PNR) during the wheat and rice growth stages**. Values are presented as mean ± SE (*n* = 3). Letters not shared across columns within one sampling time indicate significant differences (Tukey, *P* < 0.05) among four fertilizer treatments. NNF, no nitrogen fertilizer; CF, chemical fertilizer; OIMF, organic-inorganic mixed fertilizer; OF, organic fertilizer.

Soil PNR was significantly affected by plant growth and fertilization, and the effects of fertilization were more important (Table S2). The three fertilization treatments with N inputs had higher PNR than that of the NNF treatment (Figure 1). In addition, the interactive effect between these two factors on soil PNR was also significant. By using a multiple stepwise linear regression, bacterial *amoA* gene abundance was the best predictor for soil PNR explaining 48.7% (*P* < 0.001) of the PNR variation, whereas archaeal *amoA* gene abundance only accounted for 6.7% of the variation; soil properties of SOC, ${\text{NH}}_{4}^{+}$, ${\text{NO}}_{3}^{-}$, soil moisture and soil pH also played significant (*P* < 0.05) roles in the variation of PNR, which accounted for 11.5, 7.9, 1.6, 1.3, and 1.9%, respectively.

### Abundance of ammonia oxidizers

**Figure 2:**
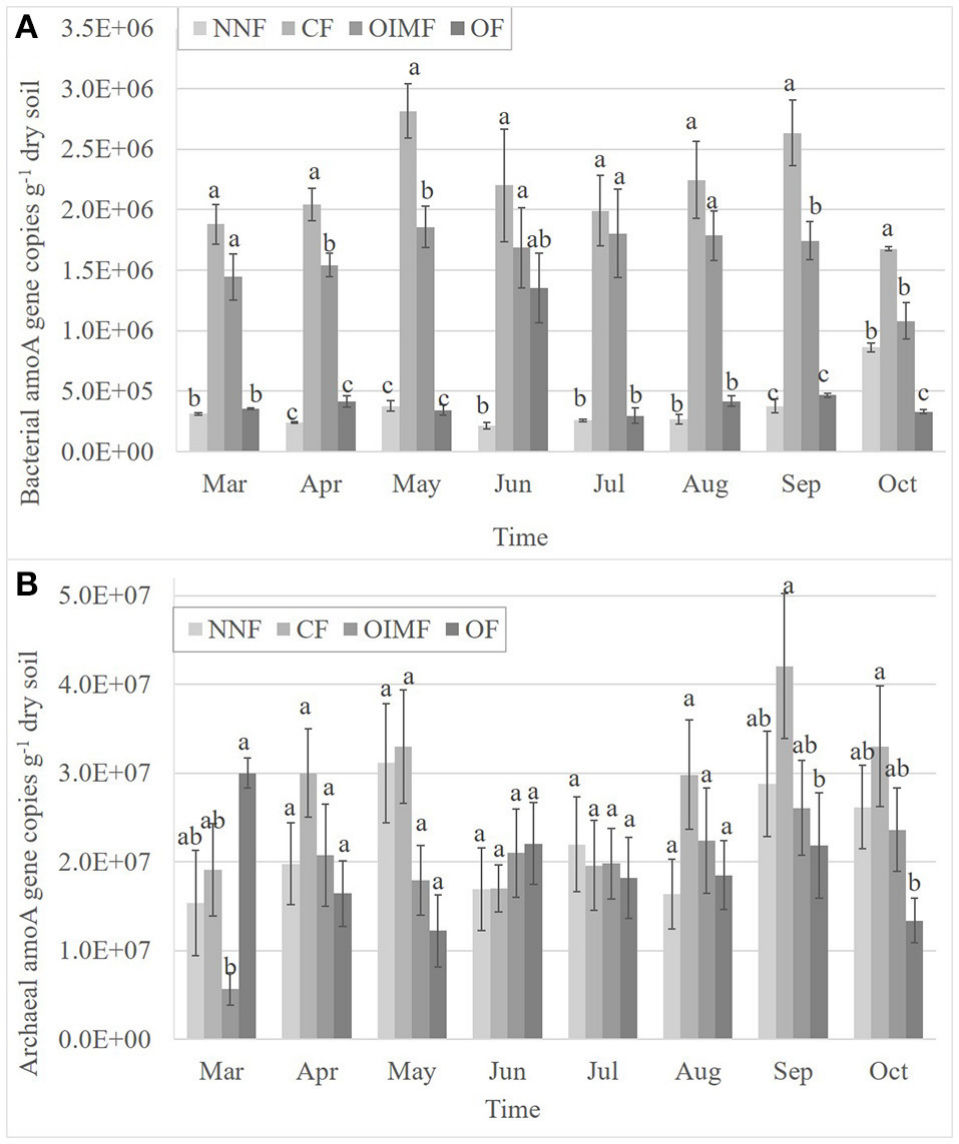
**Dynamic changes of the abundance of bacterial *amoA* gene copies (A)** and archaeal *amoA* gene copies **(B)** during the wheat and rice growth stages. Values are presented as mean ± SE (*n* = 3). Letters not shared across columns within one sampling time indicate significant differences (Tukey, *P* < 0.05) among four fertilizer treatments. NNF no nitrogen fertilizer, CF, chemical fertilizer; OIMF, organic-inorganic mixed fertilizer; OF, organic fertilizer.

As shown in Figure 2A, the abundance of AOB ranged from 2.16 × 10^5^ to 2.82 × 10^6^ copies g^−1^ dry soil, which was significantly affected by plant growth stage and fertilization and their interaction (Table S2). Compared with plant growth stage, the effect of fertilization on the AOB abundance was more important (*P* < 0.001). CF treatment had the largest AOB population sizes among the four fertilization regimes, and were 8.2 times as high as that in the NNF treatment in an average throughout all the sampling times. Although less than the CF treatment, OIMF treatment had higher AOB abundance than that of the NNF treatments across the whole stages. OF treatment had a large amount of N application (113 kg N ha^−1^), although less than that in CF and OIMF treatments (180 kg N ha^−1^), however, OF treatment had a similar low AOB abundance with NNF treatment. AOB abundance also shifted with different plant growth stages. Generally, AOB abundance increased starting at the wheat tillering stage and peaked in the wheat heading stage. This was followed by a gentle decrease in the wheat ripening and rice tillering stages. AOB abundance reached its last peak in the rice heading stage.

AOA abundance ranged from 5.71 × 10^6^ to 4.21 × 10^7^ copies g^−1^ dry soil (Figure 2B). The ratios of AOA to AOB *amoA* gene copy numbers ranged from 2 to 127 in all soil samples, indicating that the AOA abundance was higher than that of AOB. The abundance of AOA was also significantly affected by plant growth stage and fertilization and their interaction (Table S2), albeit without particular trend. For instance, OF treatment had the highest AOA abundance in Mar, but in Sep and Oct, the highest abundance was CF treatment, while in the other sampling times, there was no significant difference (*P* < 0.05) among the four fertilization treatments (Figure 2B). AOA abundance also tended to increase from the wheat tillering stage and peak in the wheat heading stage. This is followed by a gentle decrease in the wheat ripening and rice tillering stages. AOA abundance reaches its final peak in the rice heading stage. In addition, compared with the AOB (*F*-values, Table S2), both plant growth stage and fertilization had less impacts on the AOA abundance.

### Community compositions of ammonia oxidizers

**Figure 3:**
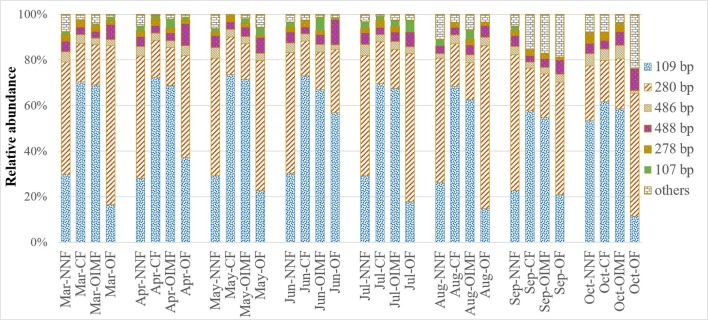
**Relative abundance of T-RFs for AOB under four fertilization regimes across eight wheat and rice growth seasons**. Three replicate T-RFs profiles of each sample were averaged into one consensus profile. NNF no nitrogen fertilizer, CF, chemical fertilizer; OIMF, organic-inorganic mixed fertilizer; OF, organic fertilizer.

As shown in Figure 3, the predominant T-RFs of the AOB community were 109 bp and 280 bp. Application of different fertilizers had a great impact on soil AOB community compositions (Table 1), while the AOB composition of CF resemble that of the OIMF (*P* > 0.01, Table S3). The relative abundance of 109 bp T-RF was significantly enhanced by CF and OIMF treatments across the whole wheat-rice rotation season. While the T-RF of 280 bp dominated in the NNF and OF treatments across the whole sampling seasons. Further phylogenetic analysis of AOB *amoA* gene revealed that 109 bp T-RF belonged to *Nitrosospira amoA* Cluster 3b, and 280 bp T-RF belonged to *Nitrosospira amoA* Cluster 3c (**Figure 5A**). Permutational multivariate analyses showed that the effect of plant growth stage on AOB community composition was slight (*P* > 0.01), we also could not detected apparent different AOB community composition of these eight plant growth stages.

**Table 1:** **Permutational multivariate analyses for the effects of fertilization regimes (Fert) and plant growth stages (Time) on bacterial and archaeal *amoA* genes T-RFLP profiles**.

	**AOB**		**AOA**	
	***R*^2^^a^**	**Pr (>F)^b^**	***R*^2^**	**Pr (>F)**
Fert	0.644	**0.001**	0.123	**0.001**
Time	0.058	0.021	0.276	**0.001**
Fert^*^Time	0.376	**0.001**	0.450	**0.001**

a *R^2^-value (effect size) shows the percentage of variation explained by the categories*.

b *Significant differences (P < 0.01) are indicated in bold*.

**Figure 4:**
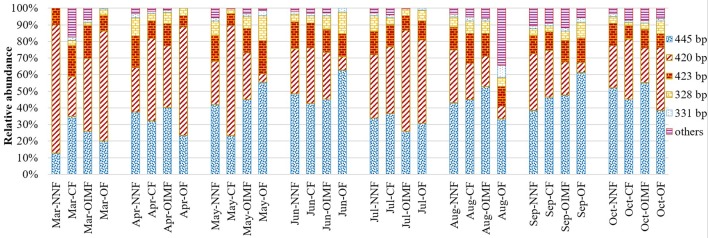
**Relative abundance of T-RFs for AOA under four fertilization regimes across eight wheat and rice growth seasons**. Three replicate T-RFs profiles of each sample were averaged into one consensus profile. NNF no nitrogen fertilizer, CF, chemical fertilizer; OIMF, organic-inorganic mixed fertilizer; OF, organic fertilizer.

**Figure 5:**
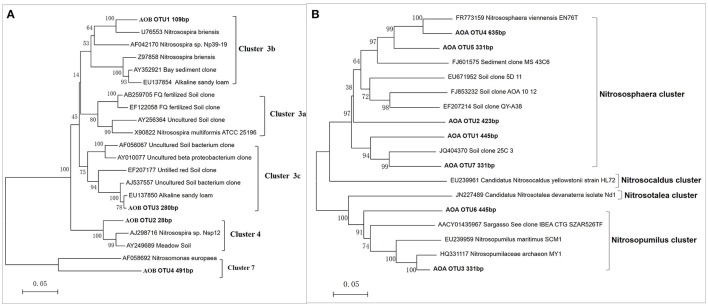
**Neighbor-joining trees based on partial bacterial *amoA* genes (A)** and archaeal *amoA* genes **(B)**. Names of sequences obtained in this study are marked in bold, the numbers in parenthesizes are their respective T-RF sizes belonging to the OTU. Bootstrap analysis of 1000 replicates values are shown next to each node.

As for the AOA community composition, the 445, 420, and 423 bp T-RFs were the dominant group, which accounted for 87% on average of the total AOA community (Figure 4). Further Phylogenetic analysis of AOA *amoA* gene revealed that T-RFs of both 445 bp and 331 bp were assigned to two clusters of *Nitrososphaera* and *Nitrosopumilus*, T-RF of 423 bp was only assigned to *Nitrososphaera* cluster (Figure 5B). Fertilization and plant growth stage significantly changed AOA community composition, by comparing the *R*^2^-values, the interaction of these two factors had the greatest influence on AOA community composition (Table 1). In contrast to the AOB composition, the effect of fertilization regime on the AOA community composition was weaker, and no clear pattern was obtained (Figure 4).

### Linking the ammonia oxidizers populations to soil properties

Correlation analysis was used to determine the relationships between the abundance of ammonia oxidizers and soil characteristics. As shown in Table 2, soil ${\text{NH}}_{4}^{+}$, ${\text{NO}}_{3}^{-}$, and the AN contents were significantly (*P* < 0.05) positively correlated with the AOB abundance, while the soil AK, soil EC and soil pH were significantly negatively correlated with the AOB abundance. For the AOA abundance, significantly (*P* < 0.05) relationship was only found with the soil ${\text{NH}}_{4}^{+}$ content. Soil PNR was significantly (*P* < 0.01) correlated with the AOB abundance but not with the AOA abundance, similar to the results of the multiple stepwise linear regression.

**Table 2:** **Spearman correlation analyses of soil properties and PNR and *amoA* gene abundances of AOB and AOA**.

	**SOC^a^**	**Soil moisture**	**${\text{NH}}_{4}^{+}$**	**${\text{NO}}_{3}^{-}$**	**AK**	**AP**	**TN**	**EC**	**pH**	**AN**	**temperature**	**PNR**
AOB	−0.101	−0.061	0.203^*^	0.348^**^	−0.537^**^	−0.085	0.035	−0.323^**^	−0.537^**^	0.414^**^	0.066	0.638^**^
AOA	0.050	0.030	0.210^*^	−0.006	−0.152	0.038	0.069	−0.194	−0.197	0.069	0.061	−0.005

a *SOC, stands for soil organic carbon; TN, stands for total nitrogen; EC, stands for electrical conductivity; AK, stands for available K; AP, stands for available P; PNR, stands for soil potential nitrification rate*.

* Indicate significant correlations at P < 0.05,

** *indicate significant correlations at P < 0.01*.

**Figure 6:**
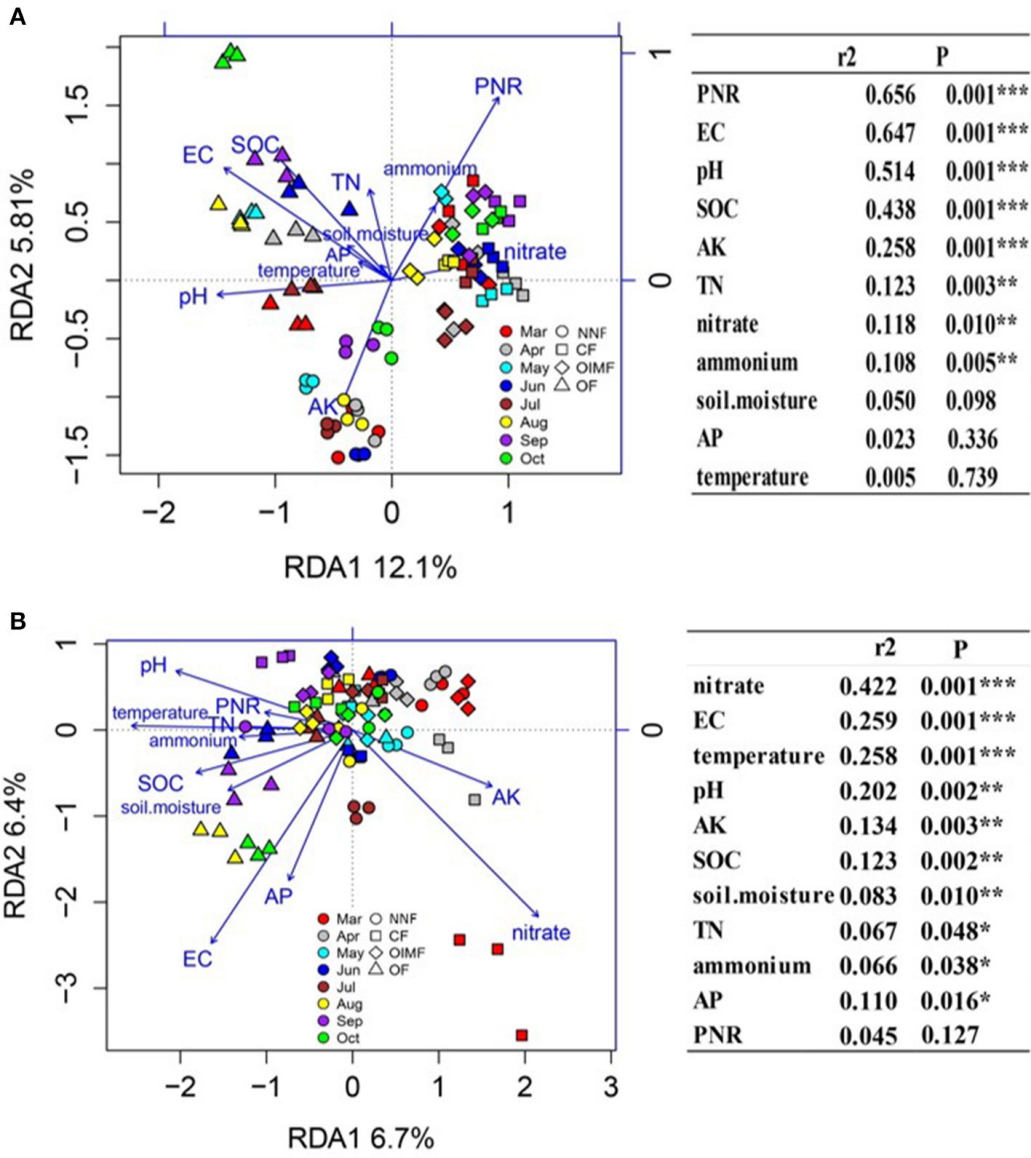
**Redundancy analysis (RDA) of the correlation of soil properties and potential nitrification rate (PNR) with AOB community structure (A)** and AOA community structure **(B)**. In the right panel, the soil properties were fitted to the ordination plots using 999 permutations test (*P*-values). Soil properties abbreviations: SOC, soil organic carbon; TN, total nitrogen; EC, electrical conductivity; AK, available K; AP, available P; NNF, no nitrogen fertilizer; CF, chemical fertilizer; OIMF, organic-inorganic mixed fertilizer; OF, organic fertilizer. ^*^ Indicate significant correlations at *P* < 0.05, ^**^ indicate significant correlations at *P* < 0.01, ^***^indicate significant correlations at *P* < 0.001.

Redundancy analysis (RDA) and permutation tests were used to determine the relationships between the soil characteristics and composition of AOB and AOA (Figure 6). The results showed that soil EC, pH, SOC, AK, TN, ${\text{NO}}_{3}^{-}$, and ${\text{NH}}_{4}^{+}$ contents were the important (*P* < 0.05) soil properties influencing the AOB community composition, and the AOA composition were driven by all the soil properties. Besides, soil PNR was significantly correlated with the AOB composition but not with that of AOA.

## Discussion

### Effects of fertilization regimes on AOB abundance and composition

Previously, we have studied the effects of fertilizations and sampling times on the total bacteria communities (Wang et al., 2016a). The results of our previous study showed that although organic fertilization (OF) tended to increase the bacteria 16S rRNA gene copies and the relative abundance of copiotrophs, plant growth stage played a central role in bacterial composition and abundance (Wang et al., 2016a). In the present study, on the contrary, fertilization regimes instead of different plant growth stages strongly influencing the abundance and composition of AOB population. These results suggested that AOB was the sensitive composition of bacteria to the change of N inputs and could be used as an indicator to soil N availability (Ceccherini et al., 2007; e Silva et al., 2013).

The addition of different fertilizers altered soil properties by changing the amount and forms of nutrients, which in turn changed the soil AOB communities. The plots receiving N fertilizers had a higher AOB abundance as compared with soil without N fertilizer, consistent with previous studies based on agricultural soils (Leininger et al., 2006; Shen et al., 2008; Di et al., 2010). Urea-N inputs increased the amount of ammonium-N in soils, which is the substrate that supplies energy for ammonia oxidizers and can be directly attributed for the increase in AOB abundance. The soils with higher amounts of ammonium-N also contained higher abundance of AOB had been reported by lots of previous studies (Jia and Conrad, 2009; Höfferle et al., 2010), the tight relationship between ammonium content and AOB abundance was also detected in present study. However, lower AOB abundance in soils treated with OIMF as compared to CF and similar AOB abundance in soils treated with OF or NNF indicated that organic-N inputting had a low impact on AOB abundance. We hypothesized that the organic-N should be mineralized into available N when utilized by AOB, but the slow release of nutrient would increase the competition between AOB and heterotrophs and plants (Lage et al., 2010). Soil pH significantly negatively influenced the AOB abundance as revealed by the correlation analysis. The relationship between soil pH and AOB abundance has been reported (Nicol et al., 2008; Bru et al., 2011; Yao et al., 2011), however, most of the previous studies were based on high pH gradient soils, whereas the pH range in our soil was only 1.5 units (6.5–8.0) showing that moderately acidic rather than moderately alkaline soil conditions are a suitable environment for AOB.

Using phylogenetic cluster analysis of the *amoA* gene, we determined that the majority of AOB were 109 and 280 bp T-RFs, which belonged to *Nitrosospira*-like Cluster 3, this result is consistent with other researches based on arable soils (Shen et al., 2008; Chu et al., 2009). In addition, our results apparently revealed that different fertilization regimes changed the AOB composition especially the relative abundance of the 109 and 280 bp T-RFs, which inferred that Cluster 3 were high variation under different fertilization treatments (Chu et al., 2007). Furthermore, the higher urea fertilization tended to have higher relative abundance of Cluster 3b (109 bp T-RF), but lower relative abundacne of Cluster 3c (280 bp). This result indicated that *Nitrosospira* 3b had a higher N demond than Cluster 3c. Previous study revealed that soil higher availiable N contents tended to has higher relative abundance of Cluster 3b (Chu et al., 2007; Shen et al., 2008), corresponding with our result. The effect of fertilizations on *Nitrosospira* 3c was not often reported, a recent research revealed that the relative abundance of Cluster 3c was dominated in the control treatment, which was higher than that in N fertilization treatments (Xue et al., 2016).

### Effects of fertilization regimes on AOA abundance and compositions

In the present study, the results of T-RFLP revealed that the predominated T-RFs were 445, 423, and 420 bp, which indicated that *Nitrososphaera* and *Nitrosopumilus* clusters were the most abundant compositions of AOA communities. As shown in Table S2 and documented by several previous studies (Santoro et al., 2008; Hai et al., 2009), the abundance and composition of AOA community tended to be insensitive to the change of environmental conditions and fertilization regimes compared with the AOB community. Additionally, there was no particular trend when we made a general survey of the archaeal *amoA* gene copies and the T-RFs profiles across the eight wheat-rice rotation seasons. These results were most likely caused by the mixotrophic lifestyle of AOA (Nicol and Schleper, 2006; Prosser and Nicol, 2012). That is, AOA could not only achieve energy from oxidizing ammonia to nitrite as autotrophs, but also assimilate the carbon and energy source from organic substrates as heterotrophs. At different sampling times, the effects of plant growth, temperature, and some other factors (Erguder et al., 2009), might have caused the AOA to alter their life strategies between autotrophic and heterotrophic, which could explain their disordered response to fertilization regimes. Indeed, the effects of fertilizations on the AOA abundance and composition were very different in previous studies. For instance, some studies revealed that the fertilizations had significant effects on AOB but not AOA abundance and composition (Shen et al., 2008; Wang et al., 2009; Wu et al., 2011), while some studies documented that AOA abundance and composition were sensitive to different fertilization regimes (He et al., 2007; Wessén et al., 2010; Chen et al., 2011). The use of only one sampling time could be the cause of these discrepant results in previous studies, it is possible that they were influenced by stochasticity and perturbations caused by varied weather conditions or human activity. Therefore, our research methodology using eight sampling times can provide a more scientific insight on the response of AOA community to fertilizations.

Surprisingly, the composition of AOA significantly correlated with all the measured soil properties showing the intensive relationships between the environmental factors and AOA composition. This was also demonstrated by other past studies based on paddy soil (Wang et al., 2014a). These results might be also be explained by the mixotrophic lifestyle of AOA, and indicate that a single parameter cannot determine the soil AOA composition (Prosser and Nicol, 2012).

### Effects of plant growth stages on ammonia-oxidizers

Different sampling times with wheat and rice growth stages also significantly influenced the abundances and compositions of both AOB and AOA. Several factors might contribute to this variation. As discussed by Erguder et al. (2009), different species of ammonia-oxidizing microbes have different optimal growth temperatures. This, coupled with the large soil temperature range in the present study, 8.9° to 31.1°C, may be partially responsible for differences in AOB and AOA abundance and composition. We also showed that soil temperature significantly effects the composition of AOA (Figure 6B). The effects of temperature on the composition of ammonia-oxidizers were also shown in a microcosm study (Tourna et al., 2008). In addition, the peak abundances of AOB and AOA were found in the most vigorous growth (head stages) of wheat and rice, which was consistent with the total bacterial 16S rRNA gene copies in our previous study (Wang et al., 2016a). This result indicated that the growth of crops could also stimulate the ammonia-oxidizers populations (Simon et al., 2000; Herrmann et al., 2008). Another important factor associated with plant growth stages was water management. From July 10th to September 30th, the rice plots were flooded with 5 cm of standing water, which might cause the depletion of soil oxygen contents. Logically, the abundance of *amoA* gene copies would decrease sharply after flooding, as described in a previous study (Fujii et al., 2010); however, we did not see a decrease in the abundance of *amoA* gene copies from Jun to Jul in either AOB or AOA. After flooding, there is an oxygen gradient established in the uppermost 2 mm of the paddy soil (Noll et al., 2005) and in a shallow zone around the roots of rice (Revsbech et al., 1999); therefore, we presume that the release of oxygen from rice rhizosphere might allow for nitrification and the growth of ammonia-oxidizers, as suggested by Liesack et al. (2000).

### Soil PNR

Quantitative PCR (qPCR) revealed that AOA had a greater amount of amoA gene copies as compared to AOB, but numerical dominance does not accurately represent functional importance (Jia and Conrad, 2009; Wang and Gu, 2013). As revealed by the multiple stepwise linear regression, correlation analysis and RDA analysis, PNR significantly correlated with the abundance and composition of AOB but not AOA, which indicated that in the period of the wheat-rice rotation system, the AOB community played a more important role in the ammonia oxidizing process than AOA. It should be emphasized that a mere correlation does not indicate the ecological importance of AOA and AOB on nitrification activities in the field (Leininger et al., 2006). DNA-based stable isotope probing proved to be a powerful tool for deciphering the ecological meaning of ammonia oxidizers in vertical profiles of paddy soils (Jia and Conrad, 2009; Xia et al., 2011). Ammonia oxidation by ammonia-oxidizers is the first and rate-limiting step of nitrification, which is a critical process in the global N-cycle (Falkowski et al., 2008). Our results indicated that inputs of organic-N had a small impact on AOB abundance, which might cause organic fertilizers to be efficient, as more of the N source will be absorbed by crops (especially the ammonium-philic crops i.e., rice) rather than oxidized by microorganisms.

## Conclusions

In summary, our results revealed that the forms of N fertilization determined the abundance and composition of AOB. The higher urea-N inputs caused higher AOB abundance, while organic-N input had less of an effect on AOB. T-RFLP of AOB revealed that CF and OIMF treatments increased the relative abundance of 109 bp T-RF (*Nitrosospira* cluster 3b) but decreased the relative abundance of 280 bp T-RF (*Nitrosospira* cluster 3c). On the contrary, the AOA were not as sensitive to fertilizations as compared with AOB. The tight relationship between PNR and AOB, but not AOA, indicated that AOB plays a dominant role in the ammonia oxidizing process of the wheat-rice rotation system. Our study provided insights into the potential of managing N for sustainable agricultural productivity in terms of soil ammonia-oxidizers.

## Author contributions

JW, LN, YS, and JL performed the majority of the experiments. JW and GR wrote the main manuscript text. QH and QS contributed insightful discussions. All authors reviewed and contributed to the manuscript.

### Conflict of interest statement

The authors declare that the research was conducted in the absence of any commercial or financial relationships that could be construed as a potential conflict of interest.
