# Supplementary material for: Dynamic Response of Ammonia-Oxidizers to Four Fertilization Regimes across a Wheat-Rice Rotation System
Source: Front Microbiol. 2017 Apr 12;8:630. doi: 10.3389/fmicb.2017.00630 (PMC5388685; doi:10.3389/fmicb.2017.00630)
Supplement: Supplementary file 1 [file Table1.DOC]

**Table S1** Soil chemical properties under the four fertilizer regimes during eight wheat-rice growth stages¤

| Stage | Fert^a^ | SOC^b^  (g kg^-1^) | TN  (g kg^-1^) | EC  (ms cm^-1^) | pH | NO_3_^-^  (mg kg^-1^) | NH_4_^+^  (mg kg^-1^) | AK  (mg kg^-1^) | AP  (mg kg^-1^) | Soil moisture  (g g^-1^) |
| --- | --- | --- | --- | --- | --- | --- | --- | --- | --- | --- |
| Mar | NNF | 17.79±0.25b | 1.88±0.07a | 0.21±0.003c | 7.43±0.02b | 3.49±0.60b | 0.00±0.00b | 181.12±14.02a | 81.42±30.8a | 0.23±0.01c |
|  | CF | 17.44±0.27b | 1.92±0.14a | 0.26±0.001a | 6.49±0.04d | 52.71±19.53a | 2.35±0.49a | 168.90±2.87a | 86.37±7.46a | 0.29±0.01b |
|  | OIMF | 16.91±0.57b | 1.77±0.21a | 0.21±0.004d | 7.19±0.16c | 8.55±1.09b | 0.13±0.13b | 126.54±6.82b | 58.46±5.52a | 0.29±0.01b |
|  | OF | 20.80±0.82a | 2.08±0.26a | 0.22±0.001b | 7.81±0.01a | 3.10±1.21b | 0.02±0.02b | 140.67±1.63b | 66.02±1.21a | 0.32±0.01a |
| Apr | NNF | 18.57±0.40c | 1.82±0.11a | 0.18±0.001b | 7.59±0.02b | 13.71±0.95b | 1.08±0.28ab | 161.83±2.50a | 65.93±8.13a | 0.27±0.01a |
|  | CF | 18.10±0.28c | 2.09±0.19a | 0.18±0.001b | 6.92±0.03d | 45.75±11.21a | 1.66±0.54a | 107.49±1.82d | 63.51±8.05a | 0.27±0.01a |
|  | OIMF | 19.77±0.17b | 2.05±0.40a | 0.17±0.004c | 7.18±0.02c | 17.58±0.81b | 1.23±0.21ab | 149.40±0.94b | 71.01±9.13a | 0.27±0.02a |
|  | OF | 21.80±0.84a | 2.21±0.24a | 0.24±0.002a | 7.87±0.05a | 11.99±0.87b | 0.72±0.17b | 130.62±3.14c | 67.7±1.19a | 0.29±0.02a |
| May | NNF | 18.48±0.23b | 2.13±0.06b | 0.24±0.001b | 7.50±0.01b | 6.06±0.24c | 0.54±0.37a | 171.25±2.04a | 67.61±3.41a | 0.24±0.01a |
|  | CF | 18.36±0.09b | 2.28±0.03ab | 0.22±0.000c | 6.88±0.03d | 12.33±0.40a | 0.40±0.08a | 102.63±2.35d | 61.28±2.62ab | 0.24±0.02a |
|  | OIMF | 19.62±0.11a | 2.44±0.11a | 0.21±0.001d | 6.97±0.02c | 6.84±0.40b | 0.70±0.10a | 156.8±2.14b | 61.29±3.46ab | 0.24±0.03a |
|  | OF | 20.20±0.56a | 2.26±0.16ab | 0.30±0.002a | 7.90±0.02a | 5.91±0.05c | 0.41±0.05a | 122.77±3.95c | 58.56±5.01b | 0.26±0.01a |
| Jun | NNF | 16.66±0.22d | 2.14±0.01a | 0.18±0.001c | 7.74±0.02b | 5.79±0.03c | 0.28±0.19b | 165.54±1.85a | 68.11±5.95a | 0.26±0.00a |
|  | CF | 17.09±0.06c | 2.16±0.09a | 0.21±0.001b | 7.11±0.04d | 22.62±0.17a | 0.65±0.07b | 115.06±0.71d | 69.78±9.12a | 0.26±0.00a |
|  | OIMF | 18.43±0.29b | 2.63±0.57a | 0.16±0.001d | 7.23±0.03c | 7.34±0.10b | 1.56±0.30a | 134.83±1.31b | 50.73±4.72b | 0.26±0.01a |
|  | OF | 20.70±0.20a | 2.1±0.66a | 0.27±0.000a | 7.90±0.06a | 7.46±0.15b | 0.97±0.66ab | 120.44±1.55c | 71.92±3.01a | 0.26±0.00a |
| Jul | NNF | 16.53±0.21b | 1.99±0.05b | 0.21±0.002b | 7.58±0.01b | 8.76±0.37b | 2.79±0.33b | 179.46±2.66a | 83.96±2.58a | 0.33±0.01a |
|  | CF | 15.55±0.00c | 1.97±0.10b | 0.18±0.001c | 7.64±0.04b | 12.64±1.03a | 6.46±1.87a | 119.19±1.94c | 77.51±0.93b | 0.33±0.01a |
|  | OIMF | 16.16±0.50b | 1.83±0.18b | 0.16±0.001d | 7.58±0.03b | 7.67±1.49b | 3.16±0.38b | 133.15±1.67b | 63.03±1.51d | 0.33±0.01a |
|  | OF | 17.87±0.09a | 2.22±0.04a | 0.25±0.001a | 7.98±0.03a | 2.38±0.48c | 5.55±1.05a | 121.73±2.34c | 68.58±1.59c | 0.32±0.02a |
| Aug | NNF | 15.45±0.41c | 1.92±0.06b | 0.22±0.023b | 7.75±0.05b | 2.10±0.28a | 2.36±0.30a | 137.56±2.85a | 63.13±3.17c | 0.30±0.01b |
|  | CF | 16.32±0.22d | 1.92±0.04b | 0.20±0.000b | 7.75±0.06b | 11.70±1.32c | 5.81±0.13b | 90.94±1.26d | 65.31±2.06bc | 0.30±0.02b |
|  | OIMF | 17.43±0.10b | 2.02±0.07ab | 0.20±0.001b | 7.65±0.03c | 6.96±1.63b | 4.98±2.66ab | 128.70±0.56b | 71.11±4.55b | 0.32±0.01ab |
|  | OF | 20.26±0.60a | 2.19±0.17a | 0.32±0.003a | 7.95±0.03a | 7.35±3.64b | 2.35±0.05b | 120.36±0.55c | 79.49±2.86a | 0.33±0.01a |
| Sep | NNF | 18.86±1.70ab | 2.14±0.16a | 0.20±0.000b | 7.55±0.03b | 1.23±0.33b | 4.54±1.31a | 163.11±1.93a | 71.84±9.99b | 0.31±0.01a |
|  | CF | 19.19±0.43ab | 2.08±0.36a | 0.17±0.001d | 7.36±0.01c | 4.44±0.08a | 3.66±0.55a | 109.13±2.15d | 75.5±2.08b | 0.32±0.01a |
|  | OIMF | 18.42±0.35b | 2.18±0.17a | 0.18±0.001c | 7.32±0.04c | 0.77±0.20c | 6.05±4.15a | 134.29±0.87b | 73.88±10.7b | 0.32±0.01a |
|  | OF | 20.67±0.86a | 2.30±0.28a | 0.31±0.006a | 7.88±0.07a | 0.49±0.25c | 3.15±0.10a | 118.79±1.46c | 90.01±7.18a | 0.33±0.01a |
| Oct | NNF | 17.54±0.26b | 1.99±0.27b | 0.20±0.002c | 7.51±0.03b | 7.00±2.17a | 4.35±1.41a | 153.58±0.79a | 57.04±5.32b | 0.26±0.01b |
|  | CF | 18.36±0.62b | 2.51±0.08a | 0.22±0.000b | 7.05±0.03c | 9.07±0.64a | 5.17±0.67a | 110.68±1.82d | 73.43±2.78a | 0.25±0.01b |
|  | OIMF | 18.00±0.81b | 2.36±0.10a | 0.20±0.003d | 7.08±0.02c | 7.17±0.78a | 5.66±0.73a | 147.06±3.23b | 64.49±5.54b | 0.27±0.02b |
|  | OF | 21.96±0.23a | 2.47±0.12a | 0.35±0.002a | 7.78±0.06a | 6.99±3.05a | 4.95±1.02a | 128.68±3.06c | 74.04±4.73a | 0.30±0.01a |

^a^ Fert stands for four fertilizer regimes: NNF no nitrogen fertilizer, CF chemical fertilizer, OIMF organic-inorganic mixed fertilizer, OF organic fertilizer.

^b^ Values are presented as mean±SE (n=3), different letters of the same sampling stage indicate significant differences among fertilizer treatments by one-way ANOVAs (Tukey, *P* < 0.05), SOC soil organic carbon, TN total nitrogen, EC electrical conductivity, AK available K, AP available P.

¤ These data were derived from our previous study

**Table S2** Two-way ANOVA for the effects of fertilization regimes (Fert) and plant growth stages (Time) on soil potential nitrification rate (PNR) and abundance of ammonia oxidizers

|  | PNR | | AOB | |  | AOA | |
| --- | --- | --- | --- | --- | --- | --- | --- |
|  | F | *P* | F | *P* |  | F | *P* |
| Fert | 236.70 | <0.001 | 378.02 | <0.001 |  | 5.38 | 0.002 |
| Time | 21.32 | <0.001 | 3.22 | 0.005 |  | 2.93 | 0.010 |
| Fert*Time | 4.26 | <0.001 | 7.57 | <0.001 |  | 2.56 | 0.002 |

**Table S3** Permutational multivariate analyses for the effects of fertilization regimes on bacterial and archaeal *amoA* genes T-RFLP profiles

|  | AOB | AOA |
| --- | --- | --- |
| NNF VS CF | 0.001*** | 0.433 |
| NNF VS OIMF | 0.001*** | 0.244 |
| NNF VS OF | 0.005** | 0.178 |
| CF VS OIMF | 0.013* | 0.157 |
| CF VS OF | 0.001*** | 0.050 |
| OIMF VS OF | 0.001*** | 0.274 |

NNF no nitrogen fertilizer, CF chemical fertilizer, OIMF organic-inorganic mixed fertilizer, OF organic fertilizer.

* indicate significant correlations at *P*<0.05, ** indicate significant correlations at *P*<0.01, *** indicate significant correlations at *P*<0.001.

Reference

Wang, J., Xue, C., Song, Y., Wang, L., Huang, Q., and Shen, Q. (2016). Wheat and Rice Growth Stages and Fertilization Regimes Alter Soil Bacterial Community Structure, But Not Diversity. Front. Microbi. 7.
